# The bottom-up effects of aridity and the top-down effects of grazing shape biodiversity across trophic levels in the Inner Mongolian grassland

**DOI:** 10.1098/rspb.2026.0561

**Published:** 2026-10-28

**Authors:** Xiaoqian Gong, Nier Su, Jia Wen, Le Ma, Biao Zhu, Qing Zhang

**Affiliations:** ^1^ School of Ecology and Environment, Inner Mongolia University, 010021, Hohhot, Inner Mongolia, People's Republic of China; ^2^ College of Urban and Environmental Sciences, Peking University, 100871, Beijing, People's Republic of China; ^3^ Ministry of Education Key Laboratory of Ecology and Resource Use of the Mongolian Plateau and Inner Mongolia Key Laboratory of Grassland Ecology, School of Ecology and Environment, Inner Mongolia University, 010021, Hohhot, Inner Mongolia, People's Republic of China

**Keywords:** aridity, diversity, grazing, grassland, soil organism, trophic cascade

## Abstract

Aridity poses a severe threat to global biodiversity, and grazing further exacerbates ecological stress in drylands. Plants and soil organisms regulate nutrient cycling and ecosystem stability through trophic cascade effects. It remains unclear whether aridity and grazing have consistent effects across trophic levels. We selected 65 paired grazing and ungrazing sites along a natural aridity gradient in the Inner Mongolian grassland in which we explored the diversity of plants, soil bacteria, fungi, nematodes and the impacts of aridity and grazing on trophic cascade effects. Increasing aridity significantly decreased the species richness of plants, pathotrophic fungi, saprotrophic fungi, herbivorous and bacterivorous nematodes. Grazing significantly increased bacterial richness and reduced the richness of herbivorous and omnivorous-predatory nematodes. Overall, aridity exerted a stronger impact on biodiversity compared with grazing. Aridity induced bottom-up effects by initially reducing plant richness, with cascading effects on soil fungal richness and higher trophic communities. Grazing reduced omnivorous-predatory nematode richness, which decreased the richness of lower trophic level organisms through top-down effects. Our research highlights different multi-trophic cascades of aridity and grazing in grasslands, emphasizing the necessity for deeper investigation into biodiversity responses across trophic levels under global change and land use.

## 1. Introduction

Grasslands cover over 40% of the terrestrial surface globally and provide essential ecosystem services and functions, including forage supply and nutrient cycling [1]. Aridity and grazing are two key factors affecting grassland ecosystems. Aridity leads to biodiversity loss in both aboveground and belowground ecosystems, severely weakening ecosystem resilience and functions [2,3]. Grazing is the dominant land use method in arid regions, altering plant and soil biodiversity through multiple pathways, including removal of plant biomass, trampling and manure inputs [4]. Exploring the effects of aridity and grazing on biodiversity simultaneously deepens understanding of the key drivers maintaining biodiversity in regional ecosystems.

Aridity and grazing can differentially influence biodiversity across trophic levels in grasslands, including plants, soil microorganisms and soil fauna [5,6]. Some studies have found that the function and stability of grassland ecosystems depend on the complex interactions across multiple trophic levels [7]. Therefore, integrating biotic communities across trophic levels is crucial for gaining a comprehensive understanding of how aridity and grazing impact grassland biodiversity. However, most existing studies focus on a single trophic level or limited biological groups. Studies that incorporate a broader range of trophic groups into a unified framework are still relatively limited [8].

Studies have found that the responses of plants and belowground organisms to environmental disturbances, such as aridity and grazing, are driven by distinct trophic cascade effects [5,9]. Variations in primary production and resource availability affect belowground microbial communities (e.g. soil bacteria or fungi) as well as herbivorous nematodes, and further regulate the diversity and functioning of higher trophic levels (e.g. omnivorous-predatory nematodes) through the bottom-up effect [10]. Conversely, the top-down effect exerted by higher trophic-level organisms can affect lower trophic levels via herbivory and predation [11]. Environmental stress and disturbance, including aridity and grazing, can influence biodiversity across trophic levels by regulating bottom-up and top-down effects, ultimately shaping the structure and stability of the whole soil micro-food web [9,12,13].

Although previous studies have revealed that both aridity and grazing can influence the soil micro-food web through bottom-up and top-down effects [5,9], their relative regulatory roles across trophic levels remain controversial. Aridity is generally considered to predominantly exert bottom-up control by decreasing plant productivity and intensifying resource limitation [14,15], but it may also influence top-down regulation by constraining the movement and activity of higher trophic-level predators and by altering biotic interactions within soil micro-food webs [9,16]. Grazing may also exert bottom-up effects by removing vegetation and altering the soil environment [5]. Meanwhile, it can influence the survival of micro-predators by physically disturbing and disrupting the soil microhabitat [17], which may regulate the prey community through top-down effects [10]. Therefore, both aridity and grazing may simultaneously influence soil trophic chains through bottom-up and top-down effects, potentially involving cross-pathway effects, although their relative importance may differ across trophic levels. However, few cross-trophic level studies have considered how these environmental stressors jointly shape multi-trophic interactions within ecosystems [18].

The Inner Mongolian grassland constitutes a critical component of the Eurasian grassland, spanning extensively and featuring a distinct aridity gradient from east to west. It offers an ideal natural platform for studying biodiversity responses across multiple trophic levels [19]. Moreover, grazing is the predominant land use in the Inner Mongolian grassland. Current studies have examined the impact of aridity and grazing on biodiversity [18,20]. However, how aridity and grazing influence plant and soil biodiversity through trophic cascade effects is still unclear. Our study explored the impact of aridity and grazing on biodiversity (plants, soil bacteria, fungi and nematodes) across multiple trophic levels, along with the interactions among trophic levels in the Inner Mongolian grassland. We hypothesize that: (i) aridity and grazing differ in the strength of their impact on biodiversity at different trophic levels, with aridity having a stronger overall effect; and (ii) aridity and grazing influence biodiversity across trophic levels through multiple pathways, with aridity exerting stronger bottom-up effects and grazing exerting stronger top-down effects. This study aims to improve our understanding of how biodiversity responds to environmental disturbances in the Inner Mongolian grassland and to inform strategies for biodiversity preservation and restoration in the context of global change.

## 2. Methods

### (a) Study area

Our study area, situated within the Inner Mongolian grassland of northern China (111°10′E-120°1′E, 41°31′N-46°45′N), experiences a temperate arid and semi-arid climate (see figure 1 in the electronic supplementary material). The region experiences 200–400 mm of precipitation and an average temperature of 1–2°C annually, decreasing from east to west. Elevation varies from 700 to 2000 m [21]. Grassland types distributed along the aridity gradient include meadow grassland, typical grassland and desert grassland. Dominant plant species include *Stipa grandis* P. Smirn., *Stipa krylovii* Roshev., *Stipa tianschanica* var. *klemenzii* (Roshev.) Norl. and *Stipa breviflora* Griseb.

### (b) Data collection

#### (i) Study design and sampling

We selected 65 pairs of grazed and ungrazed sites for vegetation and soil surveys along a nearly 700 km east–west transect during the 2022 growing season. The interval between every two sample sites was at least 10 km. We first identified ungrazed sites that had been enclosed for at least 5 years. To minimize background differences, such as climate and terrain, grazed sites were established adjacent to the corresponding ungrazed sites. Studies found that grazing intensity adjusts according to the local grassland productivity in the Inner Mongolian grassland [22,23]. Therefore, we assumed that the grazing intensities across sites were relatively consistent in this study. Both grazed and ungrazed sites had been under their respective treatments for extended periods. The Aridity Index (AI, the ratio of precipitation to potential evapotranspiration) was accessed via the Global Aridity Index and Potential Evapotranspiration Climate Database (https://cgiarcsi.community/). The aridity level in each site was characterized by the 1 – AI [6].

We randomly set up three 1 m × 1 m quadrats at each site and recorded all plant species individually. Subsequently, we collected samples from the 0–20 cm soil layer within each quadrat. Soil samples from each site were thoroughly mixed and separated into two independent subsamples: one was stored at 4°C to analyse the physicochemical characteristics, and the other at –80°C for molecular analysis of belowground communities.

### (c) Statistical analysis

To address the first hypothesis of our study, we used species richness, which is the simplest and most widely used index of biodiversity [24], as an indicator and calculated the richness of plants, soil bacteria, three fungal trophic guilds (saprotrophs, pathotrophs and symbiotrophs) and four nematode feeding groups (herbivores, bacterivores, fungivores and omnivore-predators). To explore changes in biodiversity at different trophic levels along the aridity, we conducted linear regression analyses on species richness at each trophic level in grazed and ungrazed grasslands with aridity level. Paired *t*-tests were used to assess the effects of grazing on species richness at different trophic levels. We employed ANOVA to further explore the effects of aridity and grazing, as well as their interactions, on species richness at different trophic levels. We divided sites into a semi-arid area (1 – AI < 0.8) and an arid area (1 – AI > 0.8) [6]. Before performing the variance analysis, we conducted normal distribution and homogeneity of variance tests on the dataset. Two-way ANOVA was used if the data were homogeneous in variance and had a normal distribution; otherwise, two-way non-parametric ANOVA (Scheirer-Ray-Hare test) was used. To assess grazing effects across the aridity level, we calculated the log response ratio (LRR) for each paired grazed and ungrazed site as ln [(Grazed + 1)/ (Ungrazed + 1)]. We then used linear regression to examine relationships between aridity and the LRRs of species richness at each trophic level.

To address the second hypothesis of our study, we assessed the direct and indirect effects of aridity and grazing on biodiversity at all trophic levels using structural equation models (SEMs). SEMs were constructed under the assumptions that aridity, grazing and the interaction between aridity and grazing can have both direct effects on species richness at each trophic level and indirect effects through bottom-up or top-down trophic cascades (figure 1). Based on earlier research [5,25,26], we hypothesize that aridity, grazing and their interaction can directly affect soil properties (including pH, SM, SOC, TN, NH_4_^+^-N, NO_3_^–^-N, TP and AP) and species richness at all trophic levels, and that soil regulates species richness at all trophic levels. For the bottom-up effect, plants influence soil bacteria, fungi and herbivorous nematodes [27,28], which in turn affect their predators (bacterivorous nematodes and fungivorous nematodes) [29] (figure 1a). Changes in bacteria, fungi and lower trophic-level nematodes further cascade to omnivorous-predatory nematodes [29,30]. For the top-down effect, we hypothesize that aridity and grazing further affect the species richness of low trophic levels by influencing high trophic-level organisms [9] (figure 1b). Omnivorous-predatory nematodes can regulate soil bacteria and fungi, as well as nematodes at lower trophic levels [31,32]. Bacterivorous and fungivorous nematodes further affect soil bacteria and fungi [32]. Herbivorous nematodes, soil bacteria and fungi affect plants [31]. All variables used in the structural equation models were standardized prior to analysis. Models were estimated using the robust maximum likelihood estimator (MLR), and robust fit indices were used for model evaluation. Good model fit used the following thresholds: 0.05< *p* ≤ 1.00, 0 ≤ *χ*^2^/d.f. ≤ 2, 0 ≤ root mean square error of approximation (RMSEA) ≤ 0.05, 0.97 ≤ comparative fit index (CFI) ≤ 1.00, and 0 ≤ standardized root mean square residual (SRMR) ≤ 0.05 [33]. We calculated the direct effects, indirect effects and standardized total effects (direct effects plus indirect effects) to demonstrate the responses of species richness more intuitively at each trophic level to aridity and grazing.

**Figure 1.**
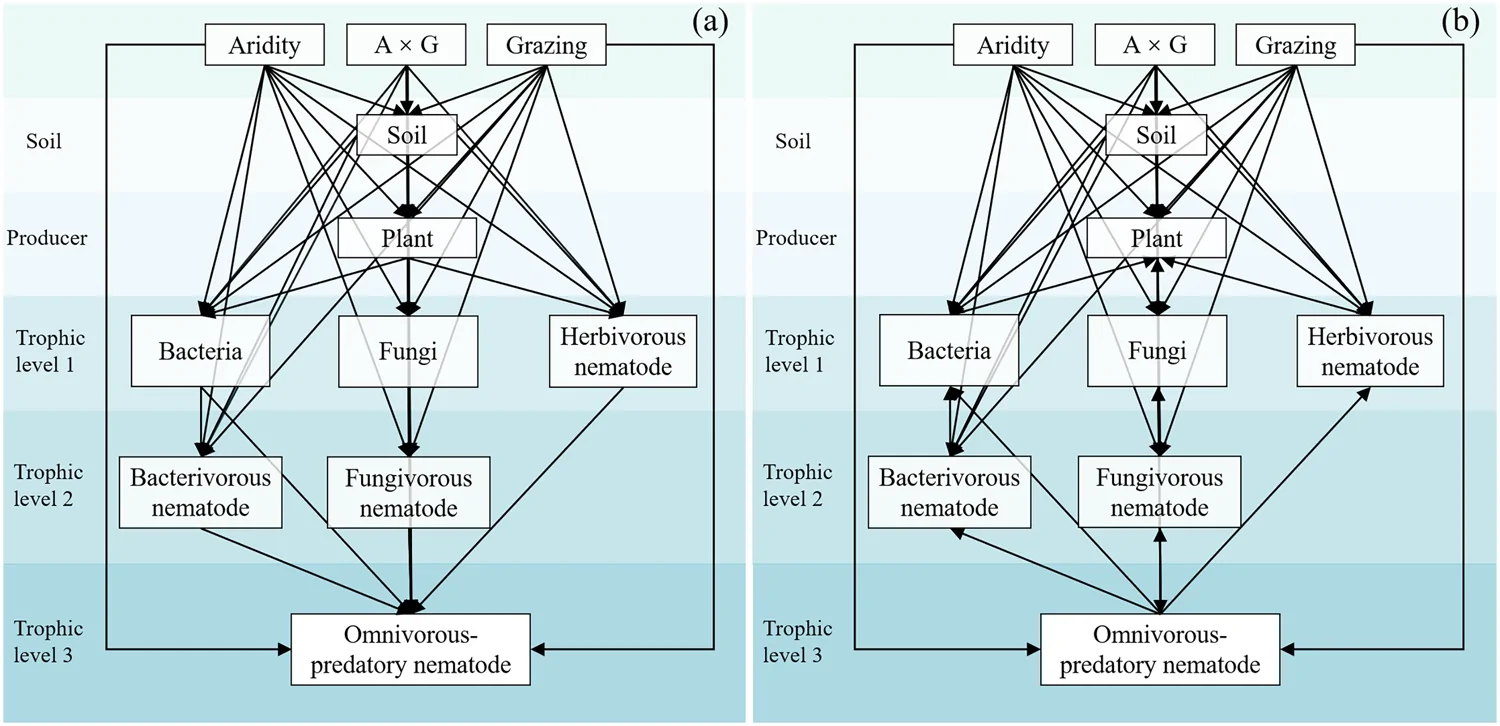
Hypothetical pathways through which aridity and grazing directly and indirectly affect the species richness of organisms across trophic levels through bottom-up (a) and top-down (b) effects. A × G represent the interaction between aridity and grazing. Arrows indicate hypothesized causal relationships between variables. Black lines represent the assumed pathways.

All data analyses were done in R v.4.3.2 [34]. The ‘vegan’ package was used to compute species richness at each trophic level [35]. We used the ‘stats’ package for linear regression analysis, two-way ANOVA and paired *t*-tests [34]. Two-way non-parametric ANOVA was performed using the ‘rcompanion’ package [36]. The ‘lavaan’ package was used to perform structural equation models [37].

## 3. Results

### (a) Effects of aridity on species richness at each trophic level

Species richness varied among trophic groups, ranging from 2–31 for plants, 1151–3421 for soil bacteria, 6–44 for pathotrophic fungi, 26–173 for saprotrophic fungi, 12–221 for symbiotrophic fungi, 2–79 for herbivorous nematodes, 2–406 for bacterivorous nematodes, 0–27 for fungivorous nematodes and 103–785 for omnivorous-predatory nematodes (figure 2).

**Figure 2.**
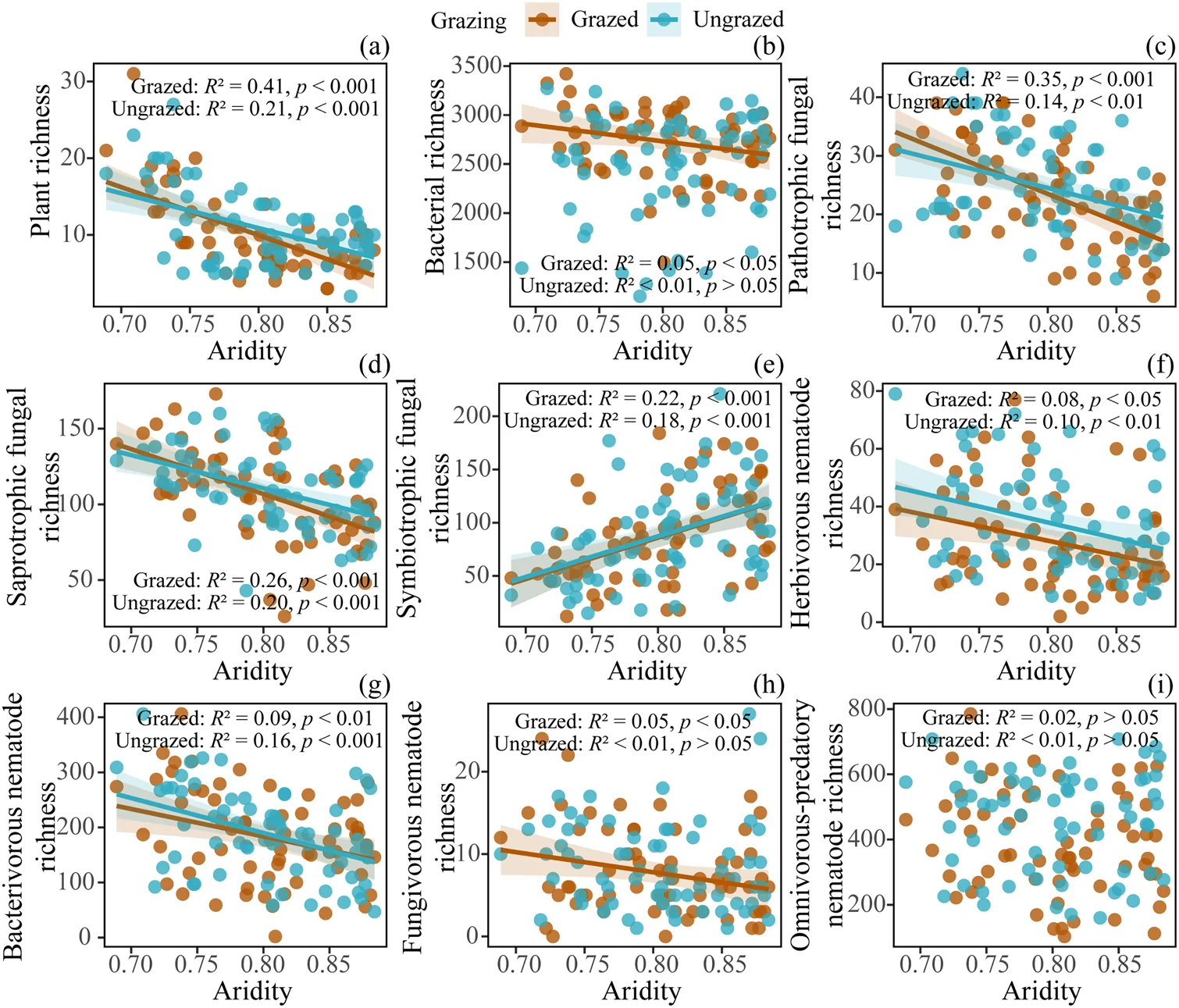
The species richness of plants (a), soil bacteria (b), pathotrophic fungi (c), saprotrophic fungi (d), symbiotrophic fungi (e), herbivorous nematodes (f), bacterivorous nematodes (g), fungivorous nematodes (h) and omnivorous-predatory nematodes (i) in grazed and ungrazed grasslands along aridity level (1 – AI). The orange dots and lines represent grazed grasslands, and the blue dots and lines represent ungrazed grasslands. The non-significant regression line is not shown. The shaded areas indicate 95% confidence intervals of the regression lines. The two-tailed statistical tests indicate significant effects at *p* < 0.05, *p* < 0.01 and *p* < 0.001.

In both grazed and ungrazed grasslands, increasing aridity significantly reduced the richness of plants (*p* < 0.001; figure 2a), soil pathotrophic fungi (*p* < 0.001, *p* < 0.01; figure 2c), saprotrophic fungi (*p* < 0.001; figure 2d), herbivorous nematodes (*p* < 0.05, *p* < 0.01; figure 2f) and bacterivorous nematodes (*p* < 0.01, *p* < 0.001; figure 2g). In grazed grassland, soil bacterial richness (*p* < 0.05; figure 2b) and fungivorous nematode richness (*p* < 0.05; figure 2h) also declined significantly with increasing aridity. By contrast, symbiotrophic fungal richness increased significantly in both grazed and ungrazed grasslands as aridity increased (*p* < 0.001; figure 2e). Omnivorous-predatory nematode richness showed no significant response to aridity (*p* > 0.05; figure 2i).

### (b) Effects of grazing on species richness at each trophic level

Grazing significantly increased soil bacterial richness (*p* < 0.001; figure 3b), but reduced the richness of both herbivorous nematodes (*p* < 0.05; figure 3f) and omnivorous-predatory nematodes (*p* < 0.05; figure 3i). Other species richness was not significantly affected by grazing (figure 3).

**Figure 3.**
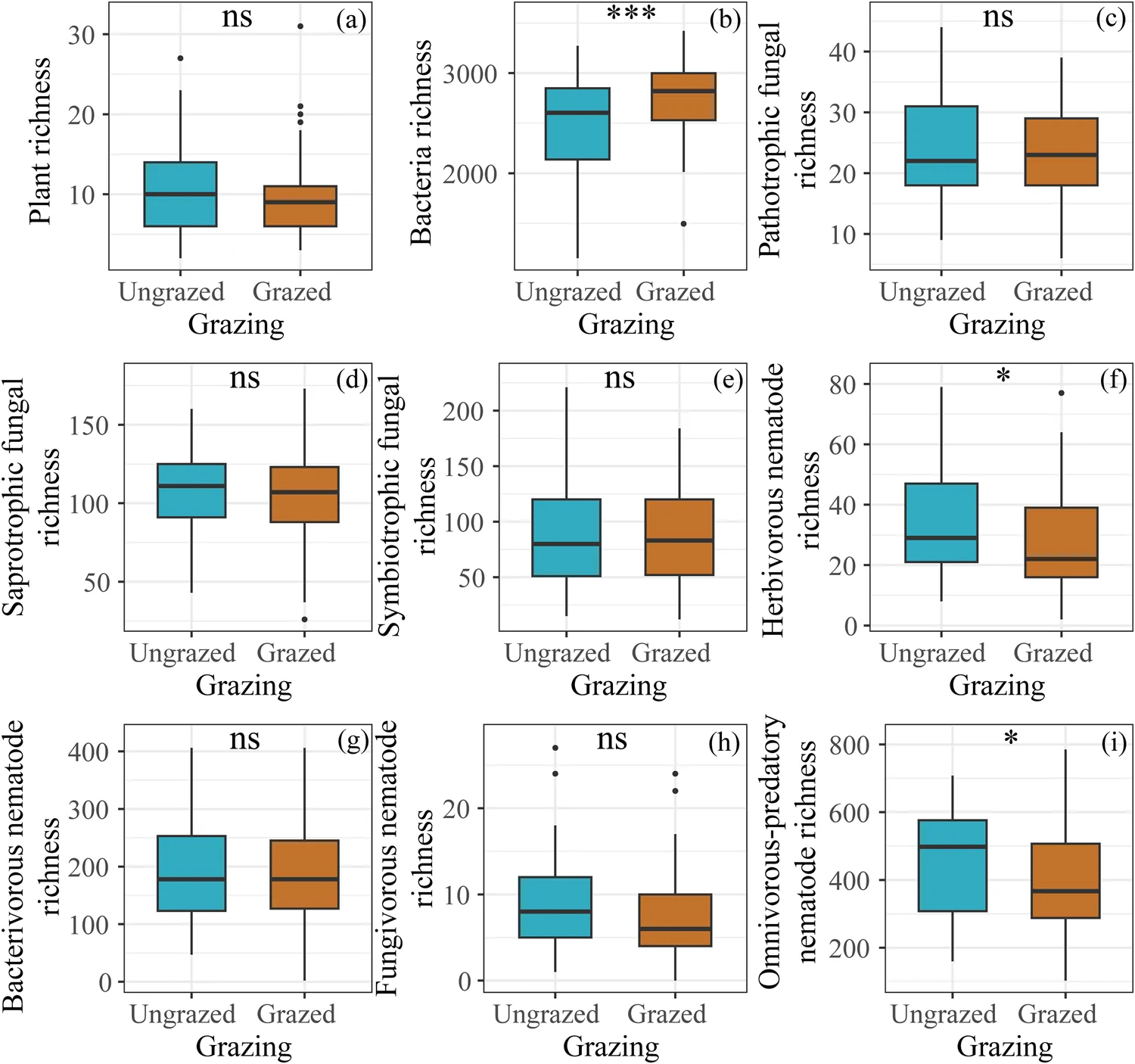
The species richness of plants (a), soil bacteria (b), pathotrophic fungi (c), saprotrophic fungi (d), symbiotrophic fungi (e), herbivorous nematodes (f), bacterivorous nematodes (g), fungivorous nematodes (h) and omnivorous-predatory nematodes (i) varies between grazed and ungrazed grassland. The blue boxes represent ungrazed grasslands, and the orange boxes represent grazed grasslands. The horizontal line inside the box represents the median; the whiskers extend to the maximum and minimum values; points outside the whiskers are outliers. The two-tailed statistical tests indicate significant effects at *p < 0.05, **p < 0.01 and ***p < 0.001. ns, not significant (*p* ≥ 0.05).

### (c) The impact of aridity, grazing and their interaction on species richness at each trophic level

The species richness of plants, soil pathotrophic fungi, saprotrophic fungi, symbiotrophic fungi, herbivorous nematodes, bacterivorous nematodes and fungivorous nematodes was significantly affected by aridity. Grazing significantly influenced the richness of soil bacteria, herbivorous nematodes and omnivorous-predatory nematodes. Plant richness was significantly affected by the interaction between aridity and grazing (table 1). Consistent with this result, regression analyses of grazing effect size (LRR) showed that the effect of grazing on plant richness became increasingly negative with increasing aridity (*p* = 0.031, figure 4a). No significant relationships between aridity and grazing effect size were observed for any other trophic group.

**Table 1.** Effects of aridity, grazing, and their interaction on species richness across trophic levels.

Variable	Factor	df	H or F statistic	*p* value
Plant richness	Aridity	1	17.276	<0.001***
	Grazing	1	1.551	0.213
	Aridity × Grazing	1	4.479	0.034*
Bacterial richness	Aridity	1	0.854	0.356
	Grazing	1	7.231	0.007**
	Aridity × Grazing	1	2.097	0.148
Pathotrophic fungal richness	Aridity	1	22.816	<0.001***
	Grazing	1	0.170	0.680
	Aridity × Grazing	1	1.215	0.270
Saprotrophic fungal richness	Aridity	1	24.772	<0.001***
	Grazing	1	0.788	0.376
	Aridity × Grazing	1	1.533	0.218
Symbiotrophic fungal richness	Aridity	1	26.147	<0.001***
	Grazing	1	0.003	0.957
	Aridity × Grazing	1	0.202	0.653
Herbivorous nematode richness	Aridity	1	18.806	<0.001***
	Grazing	1	5.523	0.019*
	Aridity × Grazing	1	0.174	0.677
Bacterivorous nematode richness	Aridity	1	19.139	<0.001***
	Grazing	1	0.206	0.651
	Aridity × Grazing	1	0.078	0.781
Fungivorous nematode richness	Aridity	1	5.567	0.018*
	Grazing	1	1.104	0.293
	Aridity × Grazing	1	0.438	0.508
Omnivorous-predatory nematode richness	Aridity	1	2.747	0.097
	Grazing	1	4.518	0.034*
	Aridity × Grazing	1	2.000	0.157

The two-tailed statistical tests indicate significant effects at *p < 0.05; **p < 0.01, and ***p < 0.001.

**Figure 4.**
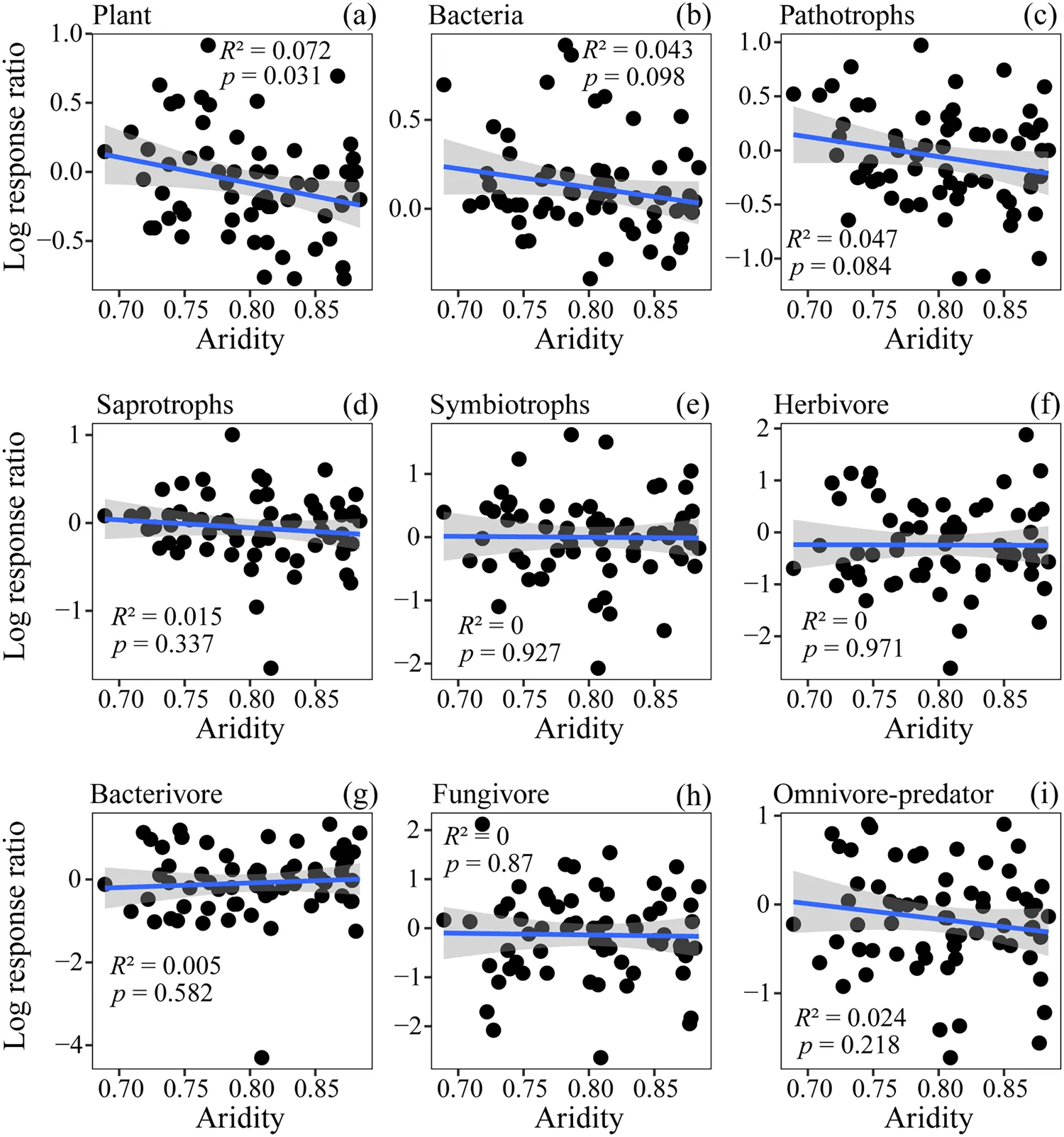
Relationships between aridity and grazing effect size (log response ratio, LRR) for plants (a), soil bacteria (b), pathotrophic fungi (c), saprotrophic fungi (d), symbiotrophic fungi (e), herbivorous nematodes (f), bacterivorous nematodes (g), fungivorous nematodes (h) and omnivorous-predatory nematodes (i). Lines indicate linear regressions with 95% confidence intervals.

### (d) Direct and indirect impacts of aridity and grazing on species richness across trophic levels

The structural equation models showed good fits, as indicated by values for *p*, *χ*^2^/d.f., CFI, RMSEA and SRMR. Aridity and grazing affected species richness across multiple trophic levels through both bottom-up effects (figure 5a,c) and top-down effects (figure 5b,d).

**Figure 5.**
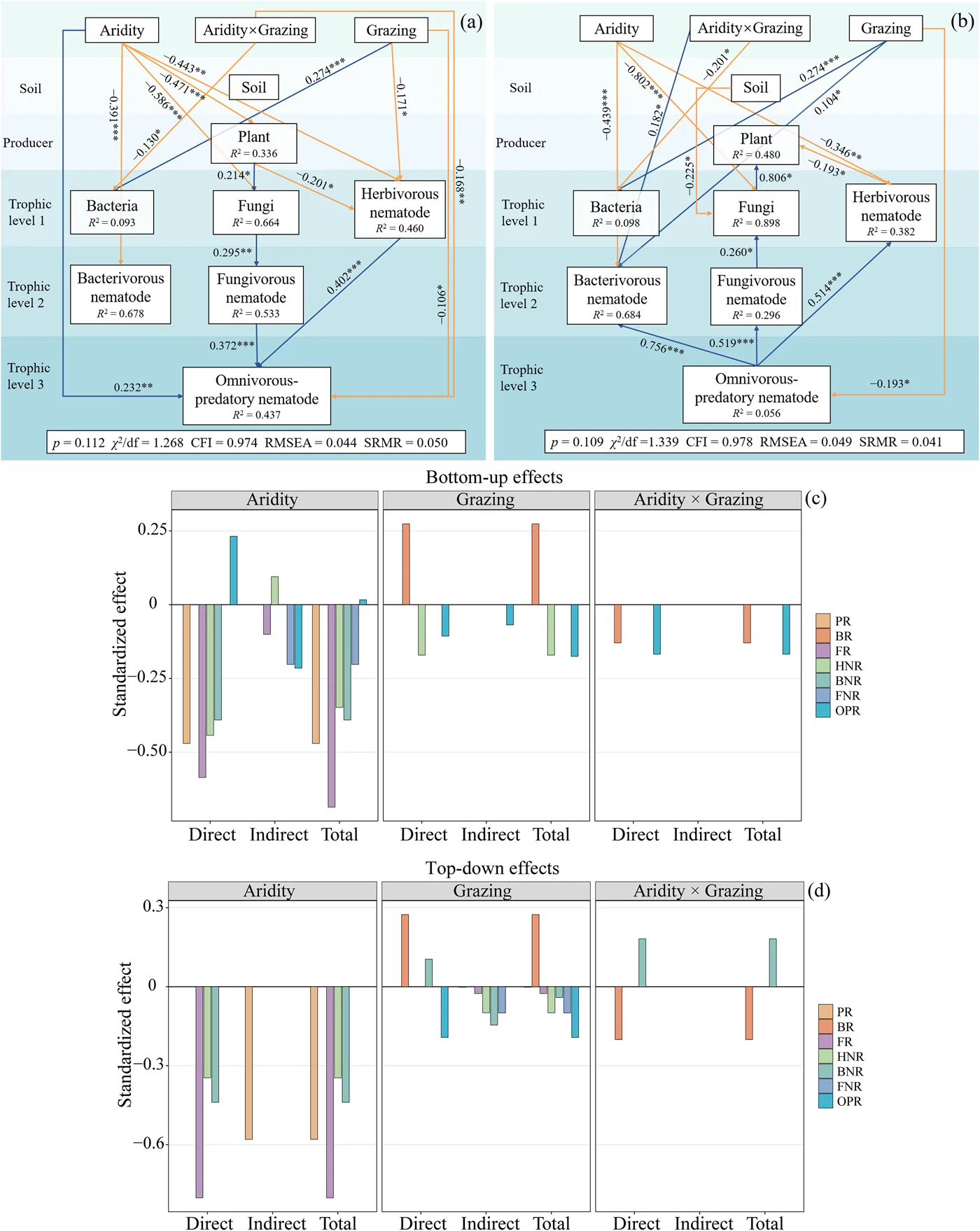
Structural equation models illustrating the direct and indirect effects of aridity and grazing on species richness across trophic levels. Panels (a) and (c) show the bottom-up effect and the standardized total effects, while panels (b) and (d) show the top-down effect and the standardized total effects. Arrows indicate the causal relationships between variables. Aridity × grazing represents the interaction between aridity and grazing. PR, plant richness; BR, soil bacterial richness; FR, soil fungal richness; HNR, herbivorous nematode richness; BNR, bacterivorous nematode richness; FNR, fungivorous nematode richness; OPR, omnivorous-predatory nematode richness. Numbers beside the lines represent the standardized path coefficients. Blue lines represent positive effects and orange lines represent negative effects. Non-significant paths are not marked. The two-tailed statistical tests indicate significant effects at *p < 0.05, **p < 0.01 and ***p < 0.001.

In the bottom-up model, aridity and grazing influenced species richness at lower trophic levels, resulting in cascading effects on higher levels through multiple pathways (figure 5a). Aridity diminished soil fungal richness either by reducing plant richness or through direct negative effects on soil fungi, which subsequently decreased fungivorous nematode richness and ultimately reduced omnivorous-predatory nematode richness. Moreover, both aridity and grazing reduced herbivorous nematode richness, which in turn further decreased omnivorous-predatory nematode richness. Aridity also indirectly increased herbivorous nematode richness through its negative effect on plant richness, although its overall effect on herbivorous nematodes remained negative (figure 5c). Overall, with the exception of soil bacteria and omnivorous-predatory nematodes, aridity negatively affected species richness at all trophic levels (figure 5a,c). Grazing negatively affected herbivorous nematode and omnivorous-predatory nematode richness, but positively affected soil bacterial richness (figure 5a,c). The interaction between aridity and grazing negatively affected bacterial richness and omnivorous-predatory nematode richness (figure 5a,c).

In the top-down model, grazing altered species richness at higher trophic levels, which subsequently influenced lower trophic levels (figure 5b). Grazing reduced omnivorous-predatory nematode richness and further suppressed herbivorous, bacterivorous and fungivorous nematode richness. Reduced fungivorous nematode richness further decreased soil fungal richness, ultimately leading to lower plant richness. Furthermore, aridity directly reduced soil fungal and bacterivorous nematode richness. Aridity indirectly decreased plant richness through reductions in soil fungal richness but indirectly increased plant richness through reductions in herbivorous nematode richness. Grazing directly increased both soil bacterial richness and bacterivorous nematode richness. Overall, aridity negatively affected the richness of plants, soil fungi, herbivorous nematodes and bacterivorous nematodes (figure 5d). Grazing positively affected soil bacterial richness but negatively affected the richness of soil fungi, herbivorous, bacterivorous, fungivorous and omnivorous-predatory nematodes (figure 5d). The interaction between aridity and grazing positively affected bacterivorous nematode richness, but negatively affected bacterial richness (figure 5b,d).

## 4. Discussion

### (a) The impact of aridity and grazing on biodiversity at each trophic level

Our findings support the first hypothesis, demonstrating that aridity and grazing differed in the strength of their effects on species richness at all trophic levels in the Inner Mongolian grassland (figure 2, table 1), and current research generally supports this result [2,25,26]. Similar to the findings of Chen and colleagues [38], aridity significantly reduced plant species richness (figure 2a). According to the carbon starvation hypothesis, drought stress can reduce plant transpiration and carbon assimilation, limiting growth and resource availability, which may ultimately reduce plant species richness [39]. Additionally, aridity can filter out drought-sensitive species, while drought-tolerant species with overlapping niches may compete more strongly under more arid conditions, collectively reducing overall plant diversity [6]. The interaction between aridity and grazing also significantly affected plant richness (table 1). The LRR analysis revealed that the negative effect of grazing on plant richness strengthened along the aridity gradient (figure 4a). Previous studies have reported that grazing and aridity interact synergistically to intensify their negative impact on biodiversity [18]. This pattern suggests that plant communities become more vulnerable to grazing under increasingly arid conditions. Therefore, appropriate grazing management may become increasingly important for maintaining plant diversity as aridity intensifies.

The responses of soil microorganisms to aridity and grazing vary. Although aridity also significantly affected bacterial richness in grazed grasslands (figure 2b), further analyses showed that grazing had a stronger overall effect than aridity (table 1; figures 3, 5). The observed increase in soil bacterial richness under grazing (figure 3b) is consistent with previous research and is probably attributable to the return of livestock excrement [40]. Grazing may also change the quantity and composition of plant root exudates, providing additional nitrogen and carbon sources that promote bacterial colonization and enhance diversity [41,42]. For soil fungi, aridity significantly reduced pathotrophic and saprotrophic fungal richness (figure 2c,d, table 1), consistent with the results of Zhang *et al.* [43]. This decline may be related to the destruction of aggregates and the reduction of organic matter driven by vegetation decline [44]. However, symbiotrophic fungal richness increased significantly with aridity (figure 2e). Plant–mycorrhizal symbiosis is considered a key strategy to cope with drought stress. Under arid conditions, plants may actively recruit symbiotrophic fungi through the insurance effect [45]. Symbiotrophic fungi enhance plant drought resistance by expanding their mycelial networks to improve water and nutrient uptake [28,46], which may account for the increased symbiotrophic fungal richness observed along aridity levels.

The impacts of aridity and grazing on nematode diversity differed markedly. Aridity significantly reduced the richness of herbivorous (figure 2f, table 1), bacterivorous (figure 2g, table 1) and fungivorous nematodes (figure 2h, table 1). Studies have demonstrated that aridity decreases soil nematode diversity [26], potentially because nematode activity is heavily dependent on soil moisture, and water scarcity disrupts their microenvironment [47]. Furthermore, grazing exerted a significant negative impact on herbivorous and omnivorous-predatory nematode richness (table 1, figure 3f,i). This result probably stems from physical soil disturbance caused by trampling, which damaged soil aggregate structure and reduced porosity [48], impairing the mobility and predation efficiency of these two nematode groups.

Furthermore, consistent with our first hypothesis, aridity had a stronger overall impact on species richness at each trophic level than grazing (figure 2). Previous studies found that the negative impact of climate change on grassland ecosystem services is higher than that of grazing [49], confirming the dominant role of aridity on the ecosystem. The greater relative contribution of aridity may be attributed to the fact that aridity induces widespread changes in nutrient cycling across trophic levels via water stress, prompting organisms to adopt ‘resource-conservative’ strategies to ensure survival [50]. However, the resource inputs resulting from faecal and urinary inputs through grazing are typically localized and lagging [5], leading to a comparatively weaker influence on overall biodiversity.

### (b) Aridity and grazing predominantly regulated different trophic cascade effects

Consistent with the second hypothesis, our study found that aridity and grazing affect the species richness of different trophic levels through different pathways (figure 5). Overall, aridity exerted its stronger effects on lower trophic levels and subsequently cascaded upward through bottom-up effects, whereas grazing primarily affected higher trophic levels and further influenced lower trophic levels through top-down effects. The dual effects modulate the interaction between plants and soil organisms in the Inner Mongolian grassland through different trophic cascades.

Aridity exerted a direct negative impact on plant richness and fungal richness (figure 5a). As previously discussed, the effect of stress during aridity was direct regulation of the physiological characteristics of plants. Aridity mediated soil fungal richness through plant richness (figure 5a), as shown in previous grassland studies [45]. The decrease in soil resource input by plants led to resource simplification and reduced fungal richness as important decomposers. The decrease in fungal richness directly restricted the food selection of nematodes that rely on fungi for food [29] and reduced fungivorous nematode richness (figure 5a). The decrease in fungivorous nematode richness, coupled with the inhibition of herbivorous nematode richness by aridity, further decreased omnivorous-predatory nematode richness at higher trophic levels through cascade effects (figure 5a) [27]. Aridity increased the richness of omnivorous-predatory nematodes overall, whereas the interaction between aridity and grazing directly reduced their richness (figure 5a,c). This result suggests that the effect of aridity on omnivorous-predatory nematodes is modulated by grazing disturbance. Under the effect of aridity, the richness of omnivorous-predatory nematodes did not show a decline, possibly owing to certain desiccation-tolerant strategies or environmental adaptation capacities of some omnivorous-predatory nematode taxa in arid ecosystems [26]. However, grazing-induced disturbance of soil microhabitats may further weaken the structure of the soil micro-food web and limit omnivorous-predatory nematodes by reducing prey availability and habitat suitability [51].

As previously discussed, grazing altered the microhabitat through livestock behaviour and directly threatened the survival and predation of omnivorous-predatory nematodes. Omnivorous-predatory nematodes occupy higher trophic levels, are larger in body size, have higher colonizer–persister- (*c–p*-) values, which represent life-history strategy ranks of nematodes, and require more resource consumption for movement and predation [51]. Therefore, they are more sensitive to the decrease in environmental disturbance and resource input caused by grazing. The reduction of omnivorous-predatory nematodes may weaken top-down control within soil micro-food webs, thereby reducing the stabilizing effects on lower trophic groups [52]. Lower trophic-level nematodes may intensify resource competition, which may ultimately reduce coexistence opportunities and richness (figure 5b) [53]. Fungivorous nematodes suppress certain fungal overgrowth through selective predation, reducing fungal richness (figure 5b) [54]. Additionally, they may reduce the number of mycorrhizal fungal hyphae through predation [55], indirectly affecting plant communities (figure 5c). The reduction of soil fungal richness weakened the ability of plants to acquire nutrients and may reduce plant richness (figure 5c) [56].

Notably, aridity indirectly enhanced herbivorous nematode richness by reducing plant richness (figure 5a). Moreover, aridity also reduced herbivorous nematode richness and further promoted plant richness (figure 5b). The opposite changes in the richness of plants and herbivorous nematodes may be explained by plant roots or root secretions being the food source for herbivorous nematodes, and they have host specificity [27,57]. Reduced plant richness may lead certain plant species to attract more specialized herbivorous nematodes through root exudates, thus increasing their richness [31]. However, the feeding activity of some herbivorous nematodes can damage plant root systems, reducing plant production and nutrient absorption [31,58]. Consequently, the reduction in herbivorous nematode richness may benefit vegetation growth and contribute to higher plant richness.

Although aridity exerted stronger bottom-up effects and grazing played a more prominent role in top-down effects, both drivers also generated cross-pathway effects (figure 5). For example, in the bottom-up effect model, grazing can also increase bacterial richness via bottom-up pathways, or reduce omnivorous-predatory nematode richness indirectly by decreasing herbivorous nematode abundance (figure 5a). In the top-down effect model, aridity can also exert top-down regulation on plant richness by affecting soil fungal richness and herbivorous nematode richness (figure 5b). This may be because grassland soil micro-food webs are shaped by the combined effects of resource availability and consumer regulation rather than a unidirectional trophic cascade [59,60]. Aridity and grazing can not only alter the resource base, such as plants and soil microorganisms, but also affect the diversity of consumers at different trophic levels [26,61]. Changes in consumer communities, in turn, can feed back to regulate resource-level organisms [62,63]. Environmental factors acting on a given trophic level often propagate along multiple pathways within the soil micro-food web, simultaneously generating both bottom-up and top-down effects [62,64]. Therefore, in grassland ecosystems, bottom-up and top-down effects are tightly coupled through soil trophic chains, jointly shaping biodiversity responses to aridity and grazing.

### (c) Limitations of this study

We investigated paired grazed and ungrazed sites along a large-scale natural aridity gradient and found that both aridity and grazing affected biodiversity across trophic levels. Overall, the effect of aridity was stronger than that of grazing. Studies that use paired-site designs to assess the effects of aridity and grazing on biodiversity under natural aridity conditions are still limited [18,65]. Our study helps to address this gap to some extent. Previous studies have shown that grazing practice and intensity can influence biodiversity [66], and inconsistent grazing intensities may lead to uncertainty in the results. However, because grasslands are owned by individual herders and the study was conducted at a relatively large scale, it was difficult to accurately quantify grazing intensity at each sampling site. We therefore classified sites as grazed or ungrazed. The rationality and effectiveness of this classification stem from the fact that a long-term grass–livestock balance policy has been implemented in Inner Mongolia since 1983. Under this policy, herders are required to adjust their livestock carrying capacity periodically, in accordance with local grassland carrying capacity, with the aim of maintaining a balance between grassland productivity and grazing pressure. For example, grazing intensity generally exceeds 2 SU ha⁻¹ in highly productive typical grasslands, but is usually below 0.5 standard sheep units (SU) ha⁻¹ in less productive desert grasslands [22]. This indicates that although the livestock carrying capacity varies in different regions, the relative use of grassland resources by livestock is comparable. Therefore, treating grazing intensity as relatively consistent across sites is reasonable in this large-scale study. Yan *et al.* [23] also adopted this method to conduct a large-scale study combining aridity and grazing in the Inner Mongolian grassland. Future studies could combine large-scale observations with networked controlled experiments to better quantify grazing intensity across grassland types, thereby allowing a more explicit assessment of the effects of aridity and grazing intensity on multi-trophic biodiversity. Furthermore, although species richness is the most commonly used and comparable measure of biodiversity across trophic levels, it captures only one dimension of biodiversity. Future research should integrate additional community attributes, including abundance, biomass and community composition, to achieve a more comprehensive understanding of the effects of aridity and grazing on grassland ecosystem structure and functioning.

## 5. Conclusions

This study reveals distinct patterns of biological responses across trophic levels in the Inner Mongolian grassland under the dual disturbances of aridity and grazing. Aridity significantly decreased the diversity of plants, pathotrophic fungi, saprotrophic fungi, herbivorous nematodes and bacterivorous nematodes, while increasing symbiotrophic fungal diversity. Grazing enhanced soil bacterial diversity but reduced the diversity of herbivorous and omnivorous-predatory nematodes. Plant diversity was significantly affected by the interaction between aridity and grazing. Overall, aridity exerted a stronger impact on biodiversity across all trophic levels than grazing. Aridity exerted stronger effects on biodiversity through bottom-up effects, primarily influencing lower trophic levels and subsequently affecting higher trophic levels. By contrast, grazing played a more important regulatory role through top-down effects, directly affecting higher trophic levels and further cascading its effects to lower trophic levels. The trophic cascade effects reveal the close connections and interactions among communities across different trophic levels in the grassland ecosystem. These findings emphasize the importance of considering dynamic relationships of communities across trophic levels in grassland management, especially under increasing aridity. Measures such as adopting optimized water resource usage and grazing management are essential to alleviate drought stress, stabilize the ecosystem structure and enhance grassland functions. Our research provides a unique perspective on the complex interactive responses of grassland ecosystems under global change and land use, offering a scientific foundation for biodiversity conservation and long-term ecosystem sustainability.

## Supplementary Material



## Data Availability

The data supporting this article can be found on Figshare [67].
