# Supplementary material for: The bottom-up effects of aridity and the top-down effects of grazing shape biodiversity across trophic levels in the Inner Mongolian grassland
Source: Proc Biol Sci. 2026 Oct 28;293(2081):20260561. doi: 10.1098/rspb.2026.0561 (PMC13725530; doi:10.1098/rspb.2026.0561)
Supplement: Supplementary file 1 [file rspb.2026.0561.zip › rspb.2026.0561/rspb20260561_si_002.pdf]

Electronic Supplementary Material

2. Methods

(a) Study area

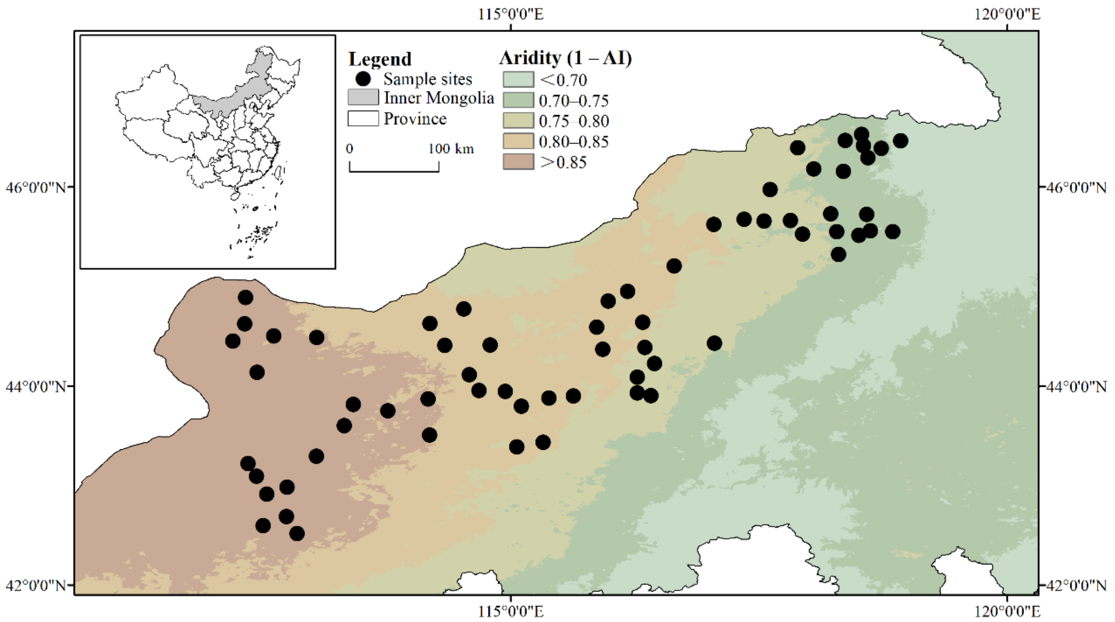

Figure 1. Location of the study area in the Inner Mongolian grassland, northern China. The main map shows the Inner Mongolian grassland, with dots indicating 65 paired sampling sites (n = 130). The inset map shows China, with the gray-shaded region representing the Inner Mongolia Autonomous Region.

(b) Data collection

(ii) Determination of soil physicochemical properties

We measured eight soil variables. Soil pH was measured using a pH meter, and soil moisture content (SM) was determined gravimetrically by oven-drying at 105°C [1]. Soil organic carbon (SOC) and total nitrogen (TN) were obtained using the $K_2Cr_2O_7$-H_2SO_4 oxidation and Kjeldahl methods, respectively [2]. Available phosphorus (AP) and total phosphorus (TP) were measured via the molybdenum-antimony colorimetric method, while ammonium nitrogen (NH_4^+-N) and nitrate nitrogen (NO_3^--N) were determined using an AA3 continuous flow analyzer [1, 3].

(iii) Molecular analysis of soil belowground communities

Soil bacterial and fungal DNA were extracted by the Cetyltrimethylammonium Bromide (CTAB) method. For soil nematodes, used the Magnetic Soil and Stool DNA Kit (DP712-02, TIANGEN, China) to extract total DNA, and quantified using Qubit (Invitrogen, USA). The primer sets (341F, 5'-CCTACGGGNGGCWGCAG-3'; 805R, 5'-GACTACHVGGGTATCTAATCC-3') were used to amplify soil bacterial DNA [4], and soil fungal DNA with primers (ITS1FI2, 5'-GTGARTCATCGAATCTTTG-3'; ITS2, 5'-TCCTCCGCTTATTGATATGC-3') [5]. PCR amplification of soil nematode DNA was performed respectively using universal primers (NF-1, 5'-GGTGGTGCATGGCCGTTCTTAGTT-3';18Sr2b, 5'-TACAAAGGGCAGGGACGTAAT-3'). PCR products were purified and quantified using AMPure XP Beads and Qubit (Invitrogen, USA). Qualified products were evaluated using the Agilent 2100 Bioanalyzer (Agilent, USA) and Illumina library quantitative kits (Kapa Biosciences, Woburn, MA, USA), and they were then sequenced on the Illumina NovaSeq 6000 (PE250), provided by LC-Bio Technology Co., Ltd. (Hangzhou, China).

Sequencing primers were trimmed from de-multiplexed raw sequences using Cutadapt (v1.9) [6]. FLASH (v1.2.8) was used to merge paired-end reads for soil bacteria, nematodes, whereas PEAR (v0.9.6) was employed for soil fungi [7, 8]. Quality control and filtering were performed with fqtrim (v0.9.4) [9]. Utilizing Vsearch (v2.3.4), chimeric sequences were filtered [10]. Amplicon sequence variants (ASVs) and their corresponding sequences were denoised and generated using DADA2. Taxonomic annotation was conducted using the NT-16S and UNITE databases for bacteria and fungi, and the NCBI databases for nematodes [11,12]. For soil fungi, we identified saprotrophs, pathotrophs, and symbiotrophs using FUNGuild, selecting guilds that were probable and highly probable with a defined single trophic mode for further analysis [13]. Nematodes were identified to the genus level and assigned to four trophic groups, including herbivores, bacterivores, fungivores, and omnivore-predators, based on feeding habits according to the Nemaplex database (http://nemaplex.ucdavis.edu) and published literature [14, 15].

References

[1] Su, N.E., Jarvie, S., Yan, Y.Z., Gong, X.Q., Li, F.S., Han, P. & Zhang, Q. 2022 Landscape context determines soil fungal diversity in a fragmented habitat. *CATENA* **213**, 106163. (doi:10.1016/j.catena.2022.106163)

[2] Su, N.E., Zhang, Q., Rineau, F., Gong, X.Q., Ding, Y. & Soudzilovskaia, N.A. 2025 Besides aridity, plant communities are a non-negligible factor in determining soil bacterial and fungal diversity. *Ecol. Appl.* **35**, e70094. (doi:10.1002/eap.70094)

[3] Yan, Y.Z., Zhang, Q., Buyantuev, A., Liu, Q.F. & Niu, J.M. 2020 Plant functional β diversity is an important mediator of effects of aridity on soil multifunctionality. *Sci. Total Environ.* **726**, 138529. (doi:10.1016/j.scitotenv.2020.138529)

[4] Logue, J.B., Stedmon, C.A., Kellerman, A.M., Nielsen, N.J., Andersson, A.F., Laudon, H., Lindström, E.S. & Kritzberg, E.S. 2016 Experimental insights into the importance of aquatic bacterial community composition to the degradation of dissolved organic matter. *ISME J.* **10**, 533–545. (doi:10.1038/ismej.2015.131)

[5] Karlsson, I., Friberg, H., Steinberg, C. & Persson, P. 2014 Fungicide effects on fungal community composition in the wheat phyllosphere. *PLoS One* **9**, e111786. (doi:10.1371/journal.pone.0111786)

[6] Martin, M. 2011 Cutadapt removes adapter sequences from high-throughput sequencing reads. *EMBnet J.* **17**, 10–12. (doi:10.14806/ej.17.1.200)

[7] Magoc, T. & Salzberg, S.L. 2011 FLASH: Fast length adjustment of short reads to improve genome assemblies. *Bioinformatics* **27**, 2957–2963. (doi:10.1093/bioinformatics/btr507)

[8] Zhang, J.J., Kobert, K., Flouri, T. & Stamatakis, A. 2014 PEAR: A fast and accurate Illumina Paired-End reAd mergeR. *Bioinformatics* **30**, 614–620. (doi:10.1093/bioinformatics/btt593)

[9] Pertea, G. 2015 fqtrim: v0.9.4 release. *Zenodo*. (doi:10.5281/zenodo.20552)

[10] Rognes, T., Flouri, T., Nichols, B., Quince, C. & Mahe, F. 2016 VSEARCH: A versatile open source tool for metagenomics. *Peerj* **4**, e2584. (doi:10.7717/peerj.2584)

[11] Nilsson, R.H., Larsson, K.H., Taylor, A.F.S., Bengtsson-Palme, J., Jeppesen, T.S., Schigel, D., Kennedy, P., Picard, K., Glöckner, F.O., Tedersoo, L., et al. 2019 The UNITE database for molecular identification of fungi: handling dark taxa and parallel taxonomic classifications. *Nucleic Acids Res.* **47**, D259–D264. (doi:10.1093/nar/gky1022)

[12] Sayers, E.W., Cavanaugh, M., Clark, K., Pruitt, K.D., Schoch, C.L., Sherry, S.T. & Karsch-Mizrachi, I. 2021 GenBank. *Nucleic Acids Res.* **49**, D92–D96. (doi:10.1093/nar/gkaa1023)

[13] Nguyen, N.H., Song, Z.W., Bates, S.T., Branco, S., Tedersoo, L., Menke, J., Schilling, J.S. & Kennedy, P.G. 2016 FUNGuild: An open annotation tool for parsing fungal community datasets by ecological guild. *Fungal Ecol.* **20**, 241–248. (doi:10.1016/j.funeco.2015.06.006)

[14] van den Hoogen, J., Geisen, S., Wall, D.H., Wardle, D.A., Traunspurger, W., de Goede, R.G.M., Adams, B.J., Ahmad, W., Ferris, H., Bardgett, R.D., et al. 2020 A global database of soil nematode abundance and functional group composition. *Sci. Data* **7**, 8. (doi:10.1038/s41597-020-0437-3)

[15] Hodda, M. 2022 Phylum Nematoda: feeding habits for all valid genera using a new, universal scheme encompassing the entire phylum, with descriptions of morphological characteristics of the stoma, a key, and discussion of the evidence for trophic relationships. *Zootaxa* **5114**, 318–451. (doi:10.11646/zootaxa.5114.1.3)
