# Supplementary material for: The bottom-up effects of aridity and the top-down effects of grazing shape biodiversity across trophic levels in the Inner Mongolian grassland
Source: Proc Biol Sci. 2026 Oct 28;293(2081):20260561. doi: 10.1098/rspb.2026.0561 (PMC13725530; doi:10.1098/rspb.2026.0561)
Supplement: Supplementary file 1 [file rspb.2026.0561.zip › rspb.2026.0561/rspb20260561_si_003.docx]

Dear Ms Erland,

Thank you very much for your careful proofreading and valuable comments. We have addressed all the suggestions point-by-point as listed below.

A) Regarding your suggestions to reduce the article length (13 pages)...Regarding reducing the page length of the article, I think that both removing **both** Sections "(ii) Determination of soil physicochemical properties" and "(iii) Molecular analysis of soil below ground communities" from the Methods section **and also** moving one figure would achieve the required result. Would this be acceptable to you? And if so, which figure would it be better to move?

*Response*: We agree to this change. We propose moving Sections "(ii) Determination of soil physicochemical properties", "(iii) Molecular analysis of soil below ground communities", and Figure 1 to the supplementary material.

B) Abstract, sentence 4, might this read better as: 'We selected 65 paired grazing and ungrazing sites along a natural aridity gradient in the Inner Mongolian grassland **in which we** explored the diversity of plants, soil bacteria, fungi, nematodes and the impacts of aridity and grazing on trophic cascade effects.'?

*Response*: We agree to this change.

C) Please edit figure 1 as follows and resupply:
i. in the top heading '**Aridity (1-AI)**', please change the hyphen to an en-rule surrounded by thin spaces, to become: '**Aridity (1** **–** **AI)**'.
ii. please replace the hyphens with en-rules in the number ranges below this, to give '0.70**–**0.75', etc**.**

*Response*: Thank you for your corrections. We have revised Figure 1 following your instructions, and the updated figure file is attached to this email for your replacement in the proof.

D) §2b(iii), last sentence of 1^st^ para, might this read better as: '**Qualified products were evaluated u**sing the Agilent 2100 Bioanalyzer (Agilent, USA) and Illumina library quantitative kits (Kapa Biosciences, Woburn, MA, USA), and **they were then** sequenced on the Illumina NovaSeq 6000 (PE250), provided by LC-Bio Technology Co., Ltd. (Hangzhou, China).'?

*Response*: We agree to this change.

E) §2b(iii), para 2, please provide a reference citation for the following:
i. 1st sentence, 'Cutadapt (v.1.9)';

*Response*: The reference citation has been prepared. The reference is given below [1]:

[1] Martin, M. 2011 Cutadapt removes adapter sequences from high-throughput sequencing reads. *EMBnet Journal* **17**, 10–12. (doi:10.14806/ej.17.1.200).

ii. 3^rd^ sentence, 'fqtrim (v. 0.9.4)';

*Response*: The reference citation has been prepared. The reference is given below [2]:

[2] Pertea, G. 2015 fqtrim: v0.9.4 release. *Zenodo*. (doi:10.5281/zenodo.20552).

iii. 5^th^ sentence, 'NT-16S', 'UNITE' and 'NCBI' databases.

*Response*: NT-16S is a curated 16S subset derived from the NCBI nt database; therefore, the NCBI reference is cited for both NT-16S and NCBI databases. The reference citation has been prepared.

The reference for UNITE is given below [3]:

[3] Nilsson, R.H., Larsson, K.H., Taylor, A.F.S., Bengtsson-Palme, J., Jeppesen, T.S., Schigel, D., Kennedy, P., Picard, K., Glöckner, F.O., Tedersoo, L., et al. 2019 The UNITE database for molecular identification of fungi: handling dark taxa and parallel taxonomic classifications. *Nucleic Acids Res.* 47, D259**–**D264. (doi:10.1093/nar/gky1022).

The references for the 'NT-16S' and 'NCBI' databases are given below [4]:

[4] Sayers, E.W., Cavanaugh, M., Clark, K., Pruitt, K.D., Schoch, C.L., Sherry, S.T. & Karsch-Mizrachi, I. 2021 GenBank. *Nucleic Acids Res*. 49, D92**–**D96. (doi:10.1093/nar/gkaa1023).

F) §2c, 2^nd^ para, sentence 2, should this be changed to: 'Based on earlier research [5, 36, 37], we hypothesize that aridity, grazing and **their** interaction can directly affect soil properties...'?

*Response*: We agree to this change.

G) The heads of the arrows in figure 2 are very small and difficult to see. Please could you prepare a new version with larger arrowheads and resupply?

*Response*: Thank you for your suggestion. We have increased the size of the arrowheads. The revised Figure 2 is attached to this email for your replacement in the proof.

H) Last para of §2c, 3^rd^ sentence, you have: 'We used the ‘stats’ package for linear regression analysis, two-way ANOVA and paired *t*-tests [45].', but reference [45] discusses the R software rather than 'stats'. Is this OK?

*Response*: Thank you for your suggestion. We propose retaining the original sentence. The 'stats' package is a core built-in package of base R. Therefore, the main R software reference (R Core Team) is appropriate here, and no separate citation for the base 'stats' package is required.

I) Reference [48] is cited at the end of the next sentence, but no year date is currently provided for this reference. Please provide one.

*Response*: We apologize for the missing information for this reference. We have supplemented the missing publication year for this entry. The reference is given below [5]:

[5] Rosseel, Y. 2012 lavaan: An R package for structural equation modeling. *J. Stat. Softw.* 48, 1–36. (doi:10.18637/jss.v048.i02).

J) Please edit figure 3 as follows and resupply:
i. In each figure part, please italicize 'R' in 'R^2^' (twice).
ii. In part (d), the U of 'Ungrazed' currently clashes with the figure outline. Please could you move the text a little to the right to avoid this?

*Response*: Thank you for your suggestion. Figure 3 has been revised: the letter R in R^2^ is italicized in both locations; the label 'Ungrazed' in panel (d) has been shifted rightwards to avoid overlapping the figure outline. The revised figure is attached to this email for your replacement in the proof.

K) Table 1:
i. Since there is plenty of space in the 'Variable' column in table 1, might it be better to spell out the variables' names in full here rather than abbreviating them?
ii. Please define 'H/F' in the column title.

*Response*: We agree that the variable abbreviations should be replaced with their full names, as the full names are provided in the table footnote. Please change “H/F value” to “H or F statistic” to clarify this column contains either H statistics (from two-way non-parametric ANOVA) or F statistics (from two-way ANOVA).

L) Figure 5:
i. In part(i) you have 'Omnivore–predators' (with an en-rule), but throughout the rest of the text this is given as 'omnivore-predator' (with a hyphen). Should the text be changed to match the figure, or *vice versa*?
ii. Throughout the figure, please edit upright 'R' to italic (9 times) and resupply.

*Response*: For (i): The label in panel (i) of Figure 5 was revised to Omnivore-predator to maintain consistency with the main text. For (ii): All nine instances of upright 'R' in R^2^ across Figure 5 have been italicized. The revised Figure 5 is attached to this email for your replacement in the proof.

M) §3d, sentence 2, should this be changed to: 'Aridity and grazing affected species richness across multiple trophic levels through both bottom-up effects ([figure 6a](https://mail.163.com/js6/read/readhtml3.jsp?mid=769:xtbDARNp3WqkhhPyEwAA3c&userType=ud&moduleName=read.ReadModule_1&font=15&color=3370FF#x_fig6),**c**) and top-down effects ([figure 6b](https://mail.163.com/js6/read/readhtml3.jsp?mid=769:xtbDARNp3WqkhhPyEwAA3c&userType=ud&moduleName=read.ReadModule_1&font=15&color=3370FF#x_fig6)**, d**).'?

*Response*: We agree to this change.

N) Figure 6: please edit as follows and resupply:
i. please italicize all 'R' in 'R^2^' and all 'χ' in 'χ^2^';
ii. in (a,b), if possible, please insert a thin space on either side of = in all *R*^2^ expressions.

*Response*: Thank you for your suggestion. The revised Figure 6 is attached to this email for your replacement in the proof.

O) §4b, para 2, sentence 2, you have: 'As previously discussed, aridity responded to stress by directly regulating the physiological characteristics of plants.'

Is it accurate to say that 'aridity responded to stress', or might it be better to say: 'As previously discussed, **the effect of stress during aridity was direct regulation of** the physiological characteristics of plants.'?

*Response*: We agree with this suggested rephrasing.

P) §4b, para 3, sentence 2, you have '*c-p*-values', but these have not been mentioned previously in the text. Is this OK, or should they be defined here?

*Response*: The *c*-*p* values are a common index for nematode life-history strategies, ranging from 1 to 5 to reflect r–K strategies; higher values indicate longer generation times and greater sensitivity to environmental disturbance. Please revise the sentence to: 'Omnivorous-predatory nematodes occupy higher trophic levels, are larger in body size, have higher colonizer–persister (*c*-*p*) values, which represent life-history strategy ranks of nematodes, and require more resource consumption for movement and predation [62].' If this definition is still unclear to readers, we are open to removing this term. Furthermore, §4b, para 3, sentence 4, please revise "omnivore-predatory nematodes" to "omnivorous-predatory nematodes".

Q) §4c, middle of the paragraph, should 'SU' be spelled out as 'stock units' at its first appearance in the text?

*Response*: SU stands for standard sheep unit, which is the standard livestock equivalent used in Chinese grassland grazing assessment. Please revise the 'SU' to 'standard sheep unit (SU)'.

R) §4c, 3^rd^ sentence from the end, might this read better as: 'Future studies could combine large-scale observations with networked controlled experiments to better quantify grazing intensity across grassland types, thereby allowing a more explicit assessment of the **effects** of aridity and grazing intensity on multi-trophic biodiversity.'?

*Response*: We agree to this change.

Our SciProof proof submission is already completed, and the system is now locked to read-only mode so we cannot upload files via the portal. The revised Figure 1, 2, 3, 5, 6 are attached to this email for your replacement. In addition, in reference 25, the first author’s name abbreviation should be changed to Su NE. No other text changes were made beyond those described in our response list. Please let me know if you require any further information or revisions.

Best regards,

Qing Zhang

[1] Martin, M. 2011 Cutadapt removes adapter sequences from high-throughput sequencing reads. *EMBnet Journal* **17**, 10–12. (doi:10.14806/ej.17.1.200).

[2] Pertea, G. 2015 fqtrim: v0.9.4 release. *Zenodo*. (doi:10.5281/zenodo.20552).

[3] Nilsson, R.H., Larsson, K.H., Taylor, A.F.S., Bengtsson-Palme, J., Jeppesen, T.S., Schigel, D., Kennedy, P., Picard, K., Glöckner, F.O., Tedersoo, L., et al. 2019 The UNITE database for molecular identification of fungi: handling dark taxa and parallel taxonomic classifications. *Nucleic Acids Res.* **47**, D259–D264. (doi:10.1093/nar/gky1022).

[4] Sayers, E.W., Cavanaugh, M., Clark, K., Pruitt, K.D., Schoch, C.L., Sherry, S.T. & Karsch-Mizrachi, I. 2021 GenBank. *Nucleic Acids Res.* **49**, D92–D96. (doi:10.1093/nar/gkaa1023).

[5] Rosseel, Y. 2012 lavaan: An R package for structural equation modeling. *J. Stat. Softw.* **48**, 1–36. (doi:10.18637/jss.v048.i02).
